# A method for creating complex real-world networks using ESRI Shapefiles.

**DOI:** 10.1016/j.mex.2023.102426

**Published:** 2023-10-11

**Authors:** Harish, Peter Mooney, Edgar Galván

**Affiliations:** a Naturally Inspired Computation Research Group, Department of Computer Science, National University of Ireland Maynooth, Ireland; b Naturally Inspired Computation Research Group, Department of Computer Science, Hamilton Institute, National University of Ireland Maynooth, Ireland; c Naturally Inspired Computation Research Group, Department of Computer Science, Hamilton Institute, National University of Ireland Maynooth, Lero, Ireland

**Keywords:** Graphical Networks, ESRI Shapefiles, NetworkX, OSMnx, Complex Real-World Networks using Geospatial Data.

## Abstract

*A classic optimization problem with many real-world applications is optimal route search in graphs or networks. Graphical networks resembling real world networks are an important requirement for these studies. Python packages* NetworkX *and OSMnx* are probably the most popular approaches in industry for creating *and analyzing real world graphical networks using ESRI S*hapefiles (Geospatial Vector Data*). However, creating such a network is a complex and tedious process as these packages require the input data to be in a specific format.* In this study,

• We outline a flexible method that can be used to easily create graphical network representations in NetworkX or *OSMnx* using road network topology data stored in ESRI Shapefiles*.*
• *A detailed step-by-step process is outlined to* successfully transform the ESRI Shapefile data into the compatible format for graph analysis libraries like OSMnx and NetworkX.
• *A data cleaning strategy is suggested to reduce resource consumption* without distorting the actual structure of the graph.

This method will allow *researchers to efficiently generate graphical networks and validate their theories by evaluating their* efficiencies *using real-world network data* of different sizes and topologies. This method could benefit, but is not limited to, research areas such as Advanced Transportation Systems (ATS), Graph Neural Networks (GNN), Multi-Objective Genetic Algorithms, to mention a few.

## Graphical abstract

**Figure:**
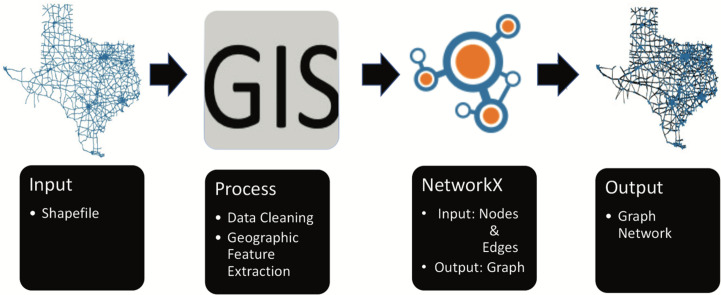

Specifications table

**Table:** 

Subject area:	Computer Science
More specific subject area:	*Graph Theory*
Name of your method:	Complex Real-World Networks using Geospatial Data.
Name and reference of original method:	1. **NetworkX:** Hagberg, A., Swart, P. and S Chult, D., 2008. *Exploring network structure, dynamics, and function using NetworkX* (No. LA-UR-08–05495; LA-UR-08–5495). Los Alamos National Lab.(LANL), Los Alamos, NM (United States). 2. **OSMnx:** Boeing, G., 2017. OSMnx: New methods for acquiring, constructing, analyzing, and visualizing complex street networks. *Computers, Environment and Urban Systems, 65*, pp.126–139.
Resource availability:	https://www.census.gov/geographies/mapping-files/time-series/geo/tiger-line-file.html

**Method details**

## Background

Path routing refers to the process of determining the optimal path between any two nodes in a graph. Because of its widespread application in areas such as transportation, logistics, and robotics, this problem has a long history in graph theory and has been extensively studied. Typically, distance is the primary objective to optimize in path routing problems. Given any two nodes *S* and *T* in a graph, Dijkstra's algorithm [1] can compute the shortest path with the complexity of O (n log *n* + m*)*. Due to its efficiency, this algorithm is widely used in applications like Apple maps, Google Maps, and others for finding the shortest path in networks.

The selection of a graphical representation can significantly influence the effectiveness of a graph theory approach. For instance, Breadth-first Search (BFS) [2] and Depth-first Search (DFS) [3] are well-known for their efficiency on small networks. However, for larger networks these algorithms become inefficient due to issues like memory limitations, time complexity, and disconnected components. Therefore, to ensure the efficiency and effectiveness of a graph algorithm, it is critical to test it on multiple networks of varying sizes and topologies.

For many years, ESRI Shapefiles have been considered as one of the most reliable sources of data for the storage and acquisition of graphical networks representing real-world road networks. An ESRI Shapefile is a vector data format that stores geospatial information about various features such as traffic signals, buildings, land parcels, roads, among others. To represent these features, they use a variety of geometries such as points, polylines, and polygons. The European Petroleum Survey Group (EPSG) is a non-profit organization that keeps and maintains geographic records using standard codes known as EPSG codes, which define the Coordinate Reference System (CRS) for ESRI Shapefile projections. The projections are stored in a binary-encoded format with a datatype called Geometry. Python packages NetworkX [4] and *OSMnx* [5] are widely known for using ESRI Shapefiles to create graphical networks. However, to convert a ESRI Shapefile representation of a network into a graph format of nodes and edges, both packages require the data to be preprocessed, cleaned, and provided in a specific format. In the following section, we detail our proposed method to take spatial data from an ESRI Shapefile containing polylines and then transform this data into the desired format for both NetworkX [4] and *OSMnx* [5].

## Method

In this study, we outline a method for transforming spatial data from an ESRI Shapefile containing polylines into the desired format for NetworkX [4] and *OSMnx* [5]. Using this method, researchers will be able to create efficient and accurate graphical representations of big and small networks to test their graph theory approaches This method will also give users a better understanding of their graphs in focus and assist them in comprehending the fundamental ideas behind graph theory, network analysis, and graph-based algorithms. To replicate this method, an ESRI Shapefile consisting of polylines in a geometry format data is required. The ESRI Shapefiles used in this study are from the open data provided by the US Census Bureau, which provides ESRI Shapefiles providing road networks for every state in the United States of America (https://www.census.gov/geographies/mapping-files/time-series/geo/tiger-line-file.html).

### Python packages

Following is the list of some useful python packages to accomplish our task. Other packages, including **OSMnx** [5] can also be used.

• *NetworkX*[4]
• *Matplotlib.pyplot*[6]
• Pandas [7]
• Geopandas [8]
• Shapely [9]

### Coordinate reference system (CRS)

The geometry objects in each ESRI Shapefile are stored using an EPSG value that defines the coordinate reference system (CRS) to accurately map the geographic location of features in the data. An EPSG code is a unique number assigned to distinct CRS. For instance, the ESRI Shapefiles used in our study have an EPSG value of 4269, which is a degree-based projection system. To calculate the distance (in meters) for all road segments in the datasets, we transformed the datasets to EPSG:2163, which is a meter-based projection system for the geographic features in the United States. Similarly, to create an accurate graphical representation from any ESRI Shapefile, it must be represented in a meter-based projection code for the geographical area. For instance, the meter-based projection code for geographic features in Australia is 3112. The database of EPSG codes may be searched at https://epsg.io/.

### Data cleaning

**Fig. 1:**
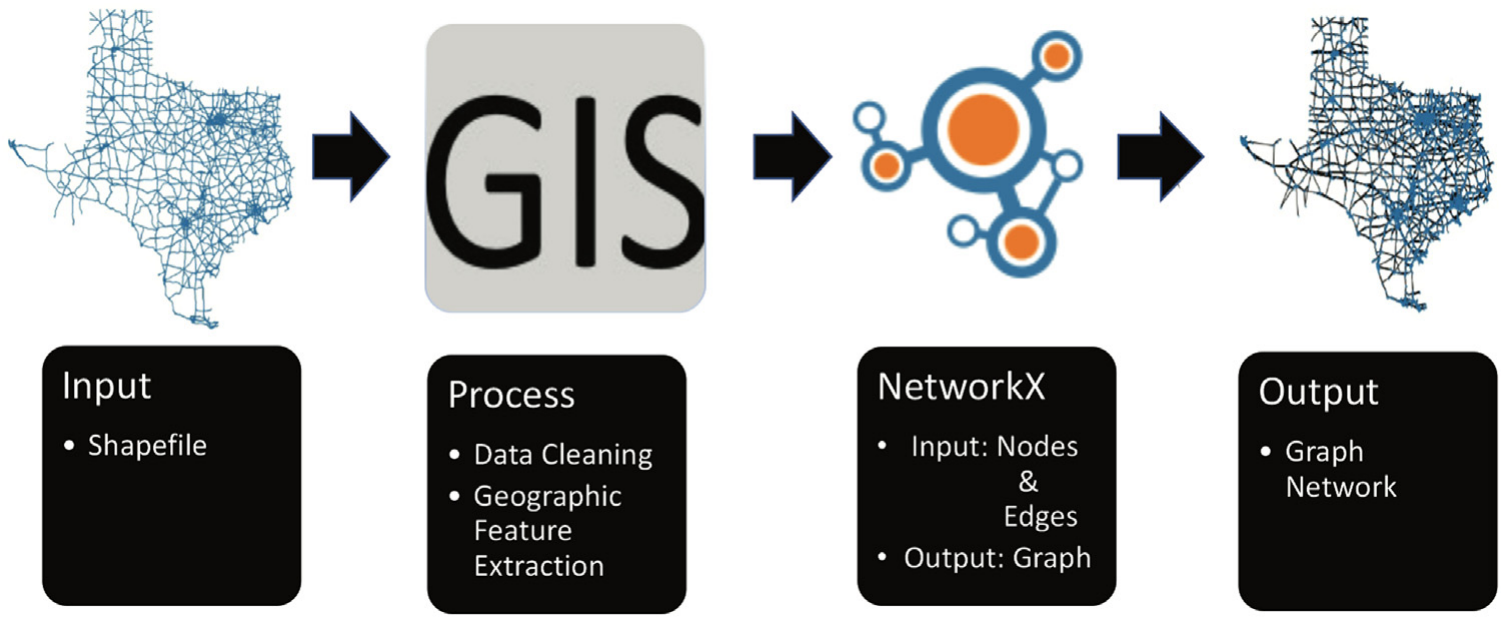
Graphical Abstract for the Process.

Many ESRI Shapefile data sources, including *OSMnx* [5] allow users to download ESRI Shapefile data for any city or country on the planet. However, this data is typically provided in raw format. Duplicate records may exist in this raw data for a variety of reasons, including representing a single feature with multiple attributes. As a result, the data must be preprocessed and cleaned before it can be used effectively (Fig. 1).

The cleaning stage described in this study not only refers to identifying and removing duplicate records but also involves identifying and removing several records that may not contribute significantly to the network. The ESRI Shapefiles used in our study contain polylines representing road segments. A polyline in our ESRI Shapefile is considered inadequate when,

• A polyline is completely contained within another polyline. For instance, an overpass represents this scenario where a new road segment is created over a pre-existing road.
• A portion (more than one consecutive point) of a polyline is contained within another polyline. For instance, city roads segments contained within major highways represent this scenario.
• Two polylines intersect with each other on more than one distinct point. For instance, consider a road that runs parallel to a highway and intersects it twice.

**Fig. 2:**
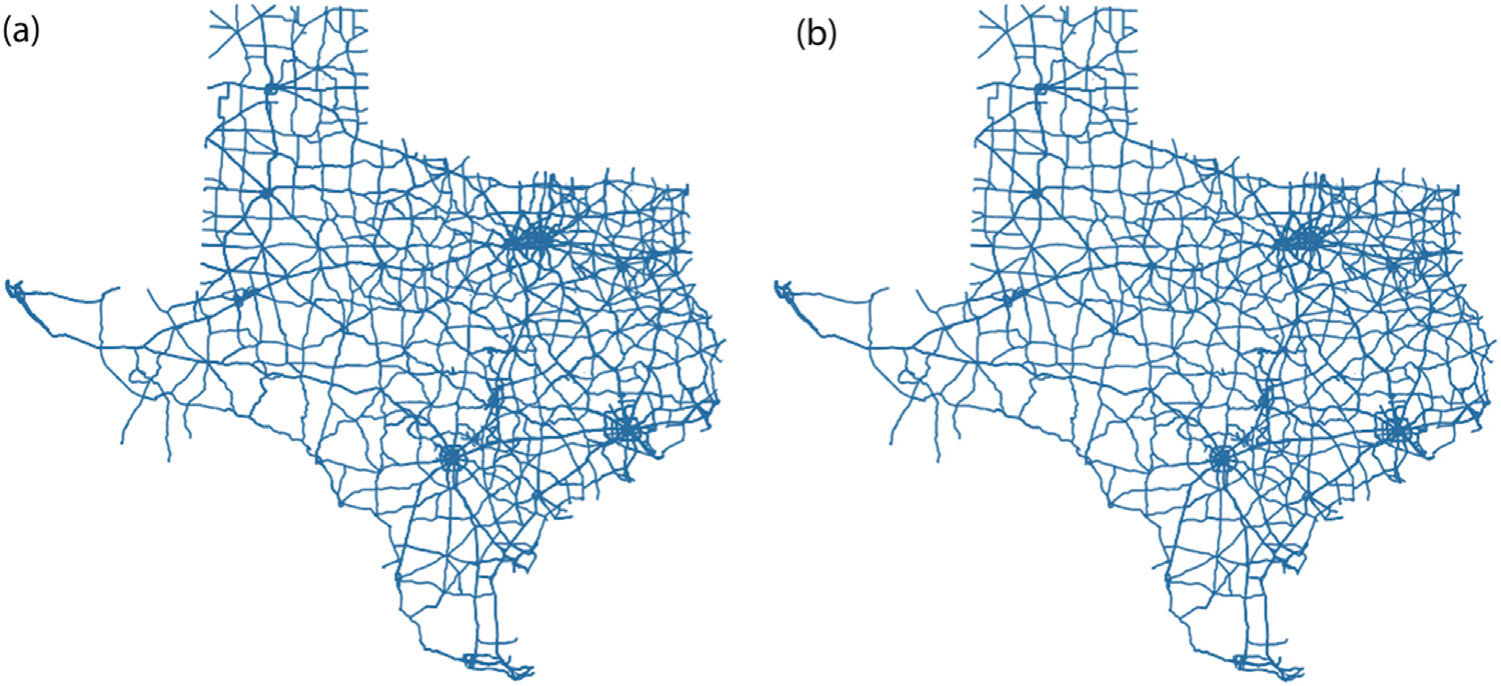
(a) ESRI Shapefile Representation of Texas before cleaning, (b) ESRI Shapefile Representation of Texas after cleaning.

Using this strategy, we can remove a considerable number of road segments from our graph without distorting the actual structure of the graph. For instance, the largest network we used in our study had 15,257 road segments. After removing the polylines complying with the aforementioned conditions, we had 7997 (only 52.4 % of the original) road segments without any considerable difference between the networks, as shown in Fig. 2.

### Geographic feature extraction

A graph is defined as a collection of vertices (nodes) and edges that connects a pair of vertices. Mathematically, a graph is defined as a pair of sets, *G* = (V, E), where V is a set of vertices and E is a set of Edges. Both NetworkX [4] and OSMnx [5] require the data in the same format to create a graphical representation of a network. In contrast to OSMnx [5] NetworkX [4] requires the user to manually extract the set of vertices and edges from the graph. While OSMnx [5] provides a convenient feature for directly extracting the required set of vertices and edges from the graph, this can lead to potential data quality issues. For our study, we extracted our data separately using python package Shapely and commonly used string manipulation techniques. Steps required to extract the unique set of vertices and edges are as followed:

1. Extract the coordinates of the first node of each polyline (Using Shapely [9]).
2. Extract the coordinates of the last node of each polyline (Using Shapely [9]).
3. Using the **intersection** property provided by python package Shapely [9], compute the polylines that share an intersecting geometry with other polylines and create a set for edges. As stated in the data cleaning strategy, two geometries sharing multiple intersections are considered invalid.
4. Extract the coordinates of all intersection nodes. It must be noted that a single polyline can share geometries with multiple unique polylines at the same or different intersection point.
5. Calculate the weight (distance) for each edge by first splitting the original polyline into two parts at the intersection node, and then calculate the length of both parts using the **split** and **length** property respectively, provided by Shapely [9].
6. At this point we now have two sets:
  a. Set of nodes containing first, last, and intersection nodes.
  b. Set of edges containing weights between two nodes.
7. Remove any duplicates from both sets.

**Fig. 3:**
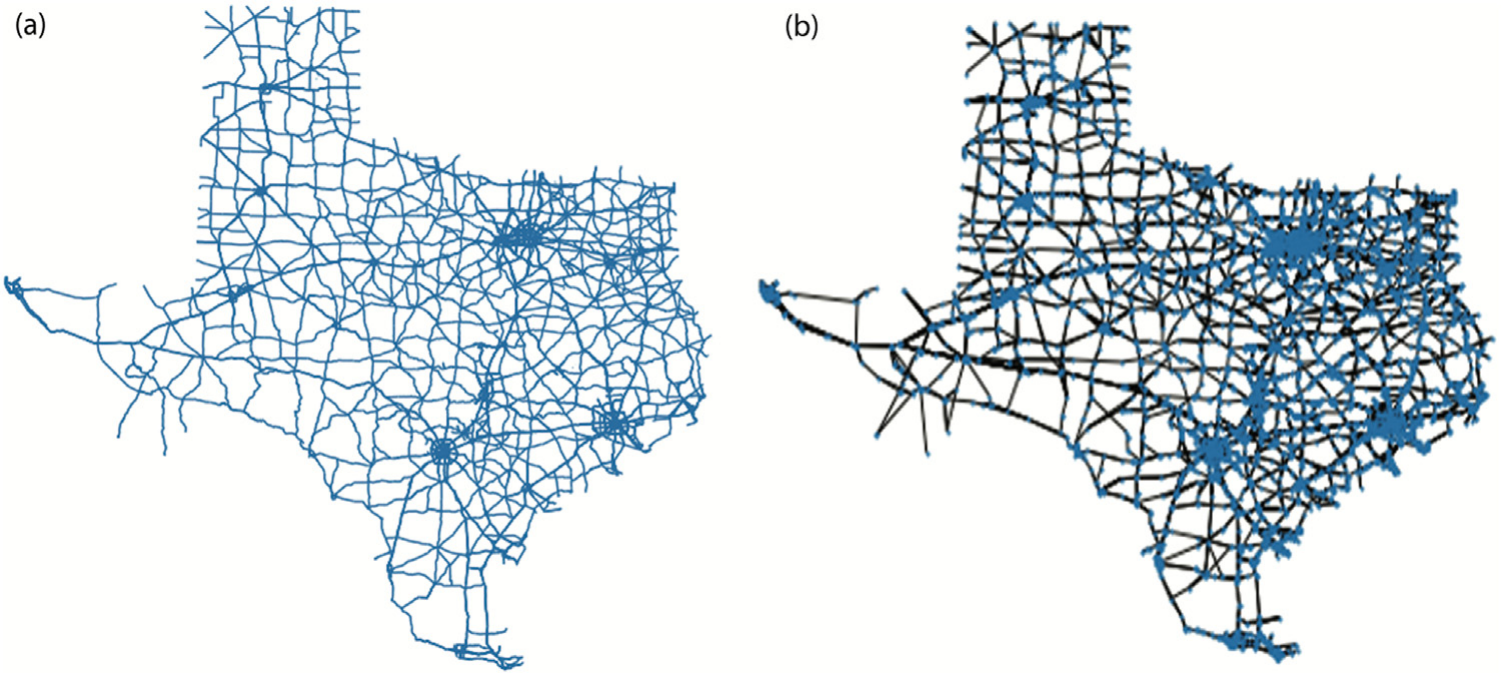
(a) Shapefile Representation of Texas after cleaning. (b) Graphical Representation of Texas using NetworkX.

Fig. 3(a) and (b) display the Shapefile Representation of Texas after cleaning and the graphical representation of Texas using NetworkX, respectively. Data cleaning steps described in Section 1.2.3 reduce the network size by 48 % but still the graphical representation obtained consists of 10,858 unique nodes and 24,407 edges.

**Fig. 4:**
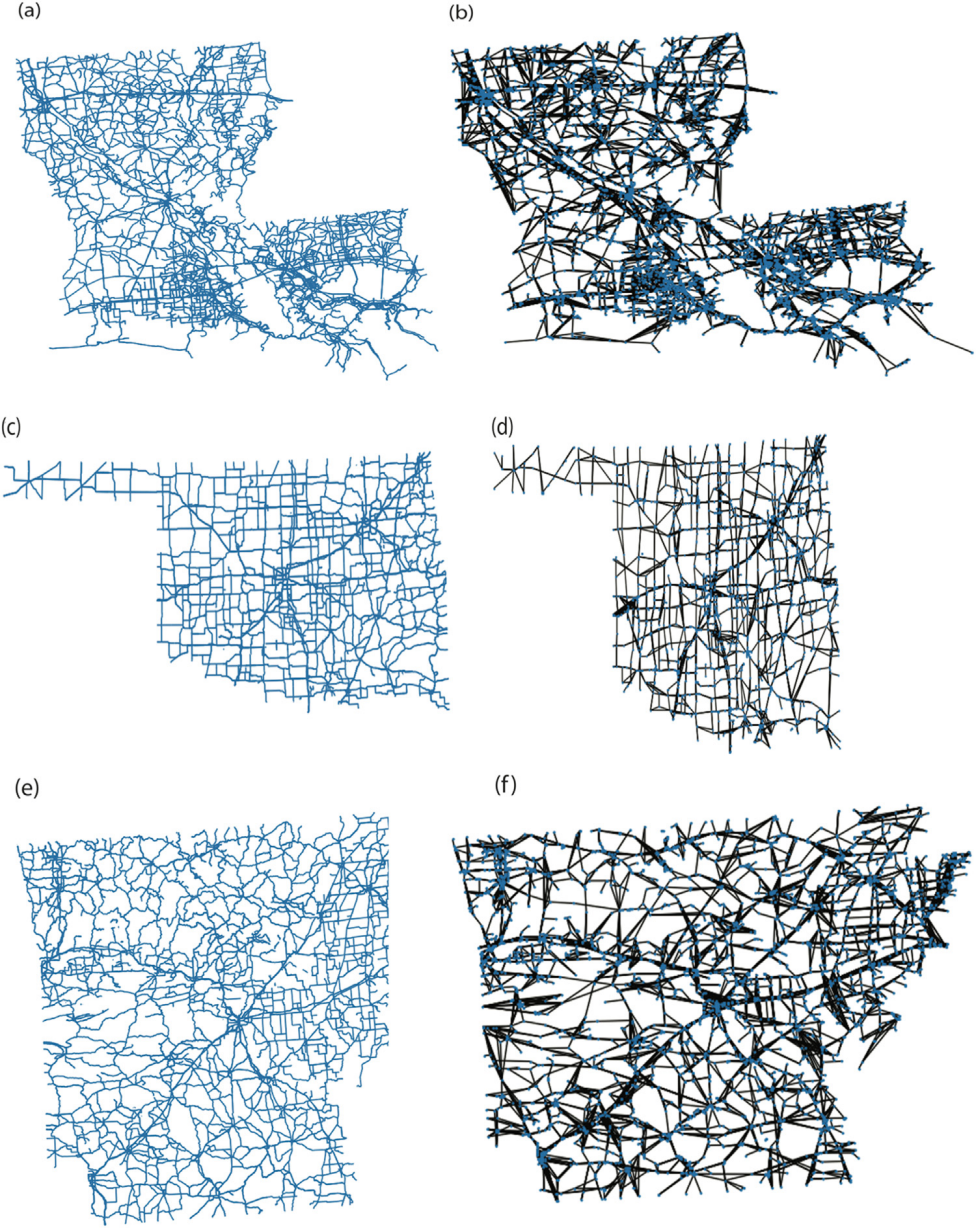
(a), (c), and (e) shows the Shapefile Representation of Louisiana, Oklahoma, and Arkansas respectively. (b), (d), and (e) displays the Graphical Representation of Louisiana, Oklahoma, and Arkansas using NetworkX respectively.

Fig. 4 illustrates the potential and reproducibility of our strategy through graphical representations of three unique networks of varying sizes. Fig. 4(a) shows an ESRI Shapefile depiction of Louisiana, which includes 9151 roadways. We were able to minimize the network size to 4616 roads, or approximately 50 % of the original size, by using the data cleaning procedure outlined in Section 1.2.3. Oklahoma's network, represented in Fig. 4(c), was the smallest of the networks investigated in our study, with 5838 roadways. Following the data cleaning, only 2743 roads (about 48 %) were deemed appropriate for investigation. The graphical representation, shown in Fig. 4(d), included 3344 distinct nodes and 6889 edges. Furthermore, Figs. 4(e) and 4(f) show the Arkansas network's ESRI Shapefile and graphical representation, respectively. The data cleaning approach mentioned in Section 1.2.3 reduced the network size from 5932 roads to 3012 roads (about 51 %), as in the prior situations. The finished graphical network had 4368 distinct nodes and 8966 edges.

## Conclusion

In this study, we proposed a method to create complex real-world networks using ESRI Shapefiles. The proposed method successfully resolves the issue of transforming ESRI Shapefile data into the necessary format for well-known graph analysis libraries like OSMnx and NetworkX. The proposed method enables researchers to quickly create graphical networks for their research projects, allowing them to test their theories on actual networks. The method's simplified data format avoids the need for time-consuming data preprocessing, allowing researchers to focus more on the analysis itself. The data cleaning strategy described in this study not only reduces resource consumption but also enhances the user's understanding of graph networks. As proof of its efficacy, the proposed method was successfully used to convert the ESRI Shapefile data from Texas, Louisiana, Arkansas, and Oklahoma into the graphical representations of the networks. As a result, this study significantly advances the field by empowering other researchers to evaluate the efficacy of graph algorithms.

## Ethics statements

We made use of open-data from the US Census Bureau (https://www.census.gov/geographies/mapping-files/time-series/geo/tiger-line-file.html), which offers ESRI Shapefiles containing road networks for all states in the United States.

## Credit author statement

**Harish:** Conceptualization, formal analysis, investigation, data curation, software, methodolog, visualization, writing – original draft, writing – review and editing. **Peter Mooney:** Supervision, writing – original draft, writing – review & editing. **Edgar Galván:** Supervision, writing – original draft, writing – review & editing. All authors have agreed to the publication of this manuscript.

## Declaration of Competing Interest

The authors declare that they have no known competing financial interests or personal relationships that could have appeared to influence the work reported in this paper.

## Data Availability

Data will be made available on request.

## Appendix Supplementary materials

Image, application 1
