# Supplementary material for: A method for creating complex real-world networks using ESRI Shapefiles
Source: MethodsX. 2023 Oct 11;11:102426. doi: 10.1016/j.mex.2023.102426 (PMC10587512; doi:10.1016/j.mex.2023.102426)
Supplement: Supplementary file 1 [file mmc1.docx]

**Supplementary material *and/or* additional information**

- Like OSMnx, the following procedure can also be performed using an application called QGIS, allowing users to extract information through a GUI. However, for precision we implemented the entire process in Python. One alternative to this approach could be using the combination of PostGIS (an open-source software for spatial data in PostgreSQL) for feature extraction and Python (NetworkX or OsMnX) for graph creation.
- Our simulation studies revealed that certain methods, such as the shortest path algorithm from the Python Package NetworkX, are only efficient for small networks. As a result, it emphasizes the significance of testing on larger real-world networks.
- The method proposed in this study can also be used to create much larger networks by combining the geographic data from multiple ESRI Shapefiles as shown in Figure 5, where we combined the geographic data from Texas, Louisiana, Oklahoma, and Arkansas using the Geopandas [8] capabilities.
- By assigning specific attributes to specific nodes, we can convert our graphical representation into a test scenario for advanced transportation system, with the aim to optimize on multiple objectives, as shown in Figure 5.

**
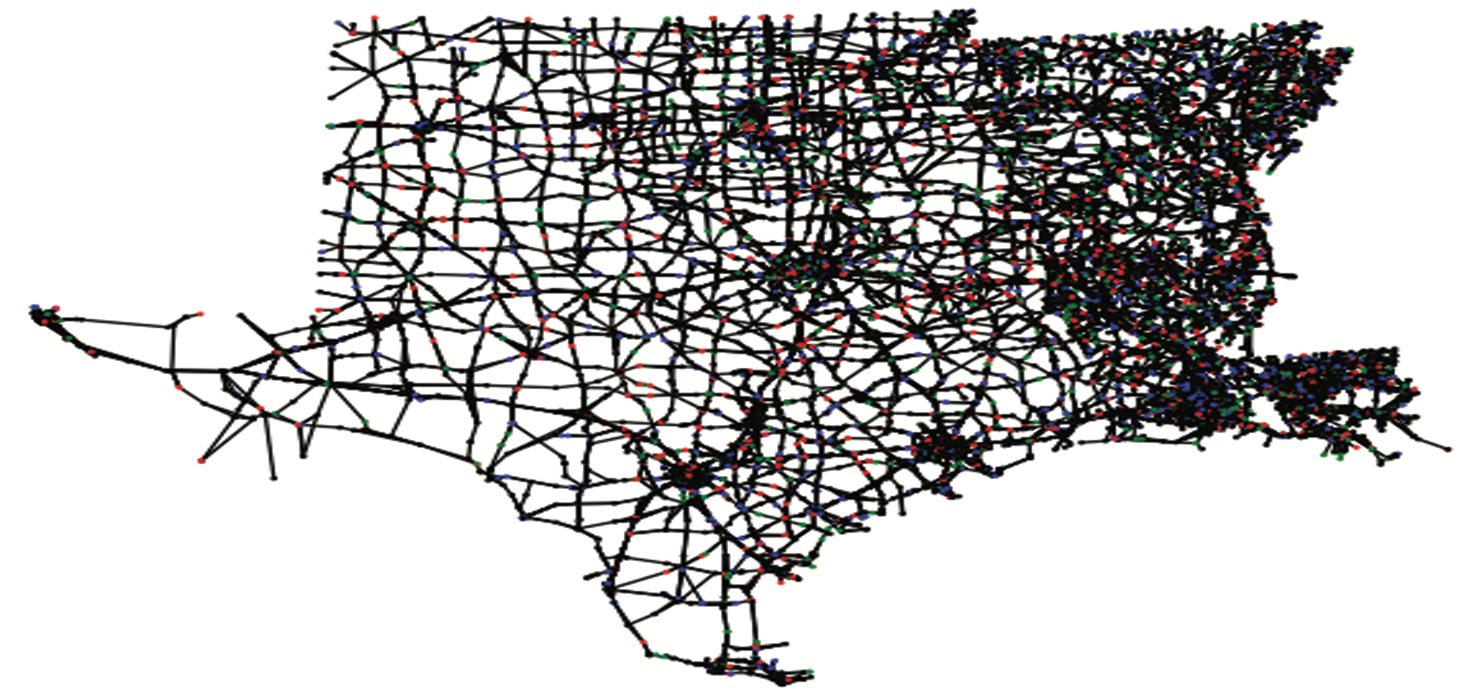
**

Figure 5: Graphical Representation of Texas, Louisiana, Arkansas, and Oklahoma as a single graph with multiple amenities represented using different color nodes.
